# Falling Off the Growth Curve: An Underrecognized Risk of Automated Formula Machines

**DOI:** 10.7759/cureus.102087

**Published:** 2026-01-22

**Authors:** Evan S Baker, Neemesh Desai, Lucas McKnight

**Affiliations:** 1 Internal Medicine-Pediatrics, The Ohio State University College of Medicine, Columbus, USA; 2 Internal Medicine-Pediatrics, The Ohio State University Wexner Medical Center and Nationwide Children's Hospital, Columbus, USA; 3 Internal Medicine, Johns Hopkins Health System, Baltimore, USA; 4 Internal Medicine, The Ohio State University Wexner Medical Center and Nationwide Children's Hospital, Columbus, USA

**Keywords:** automated formula machines, failure-to-thrive, formula, formula-fed, growth curve, growth faltering, manual vs automation, nutrition, nutrition assessment, poor weight gain

## Abstract

Growth faltering, falling off the growth curve, or failure to thrive are all common terms used to describe a frequent but clinically important scenario routinely seen in pediatrics, often identified during infancy and other periods of rapid growth. Inadequate intake is the most common cause identified, especially in the neonatal period. This can lead to lasting consequences for cognitive potential and final adult height. We present the case of a four-month-old female infant who experienced a sudden decline from the 13.6th to the 4.6th percentile in weight for age between six weeks of age and her four-month well visit. Parents reported an adequate intake volume of 32 oz/day prepared with an automated formula machine and age-appropriate elimination. A careful nutrition history regarding formula preparation revealed that the machine was set to a concentration significantly lower than recommended for her specific formula. This meant the prepared formula provided substantially fewer than the recommended 20 kcal/oz. After two weeks of manual formula fortification, the patient demonstrated improved weight gain and returned to the 14.72nd percentile in weight for age.

While growth faltering is common, this report highlights a less frequently recognized cause of inadequate intake, namely, improperly concentrated formula prepared by an automated mixing machine. This underscores the importance of a thorough pediatric nutrition history, including a detailed assessment of formula mixing methods. Physicians must be prepared to counsel caregivers on correct formula preparation, whether using manual or automated mixing methods. With automated machines, providers should consider verifying that the settings are appropriate for the specific formula. When patients present with inadequate growth, it is important to consider incorrect formula preparation as a potential etiology and to investigate further.

## Introduction

During early infancy, growth is closely monitored at pediatric visits to ensure that children are receiving adequate nutrition and growing appropriately. The newborn period is particularly crucial, with infants gaining about 28 g per day before slowing to about 20 g per day around four months of age [1]. According to the American Academy of Pediatrics, growth faltering is often due to inadequate energy supply, high energy expenditure, or a combination of the two [2]. In the neonatal period, the most common cause is inadequate intake, particularly due to insufficient breast milk supply, poor latching, or improperly prepared formula [3]. Formula-fed infants have been shown to gain weight more rapidly than breastfed infants, but improper formula preparation places them at an equally high risk of growth faltering [4]. This can have significant long-term effects if not diagnosed and treated in the first two years of life, especially regarding cognitive potential and maximal adult height [3].

## Case presentation

**Figure 1:**
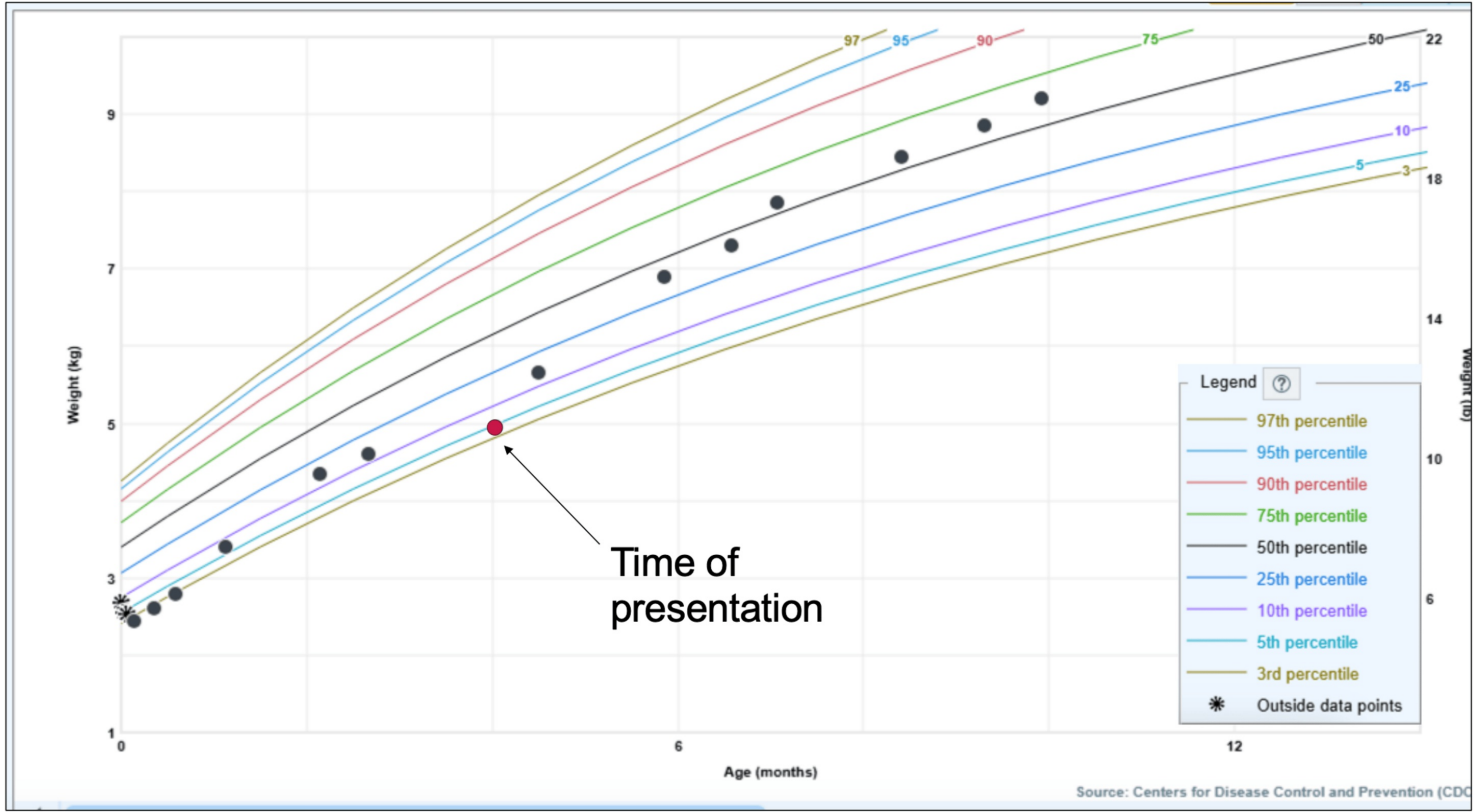
Weight-for-age percentiles (girls, birth to 36 months) Patient weight-for-age percentiles plotted on the CDC's standard growth chart for girls from birth to 36 months. Graph created by Epic Health Systems (Electronic Health Record). The patient was born at 37 weeks (term), so chronological age was used, and the CDC’s standard growth chart was appropriate CDC: Centers for Disease Control and Prevention

A four-month-old female with a history of in utero syphilis exposure and macrocephaly with a previously normal head ultrasound presented for her four-month well visit. Before this visit, she had been steadily tracking above the 10th percentile on the weight-for-age curve (Figure 1). Her parents reported feeding her commercial formula, measured as 5 oz every four to six hours. Overall, she was receiving 32 oz per day, prepared using an automated formula machine. She otherwise exhibited normal, age-appropriate elimination behaviors.

On exam, she weighed 4.955 kg, representing a 7% increase from 4.6 kg at her visit six weeks ago. Over this time, she had an average weight gain of 8.5 g/day and declined from the 13.6th to the 4.6th percentile (Table 1). She was otherwise well appearing with stable macrocephaly and bilaterally lengthened feet. Her physical exam was otherwise normal, with normal cardiac and pulmonary sounds and a normal abdominal exam. Parents denied any recent symptoms, including fevers, vomiting, or diarrhea. Given a reassuring history, exam, and prior appropriate growth, there was low concern for systemic disease, including chronic infection, metabolic disorders, or chronic cardiac, pulmonary, or renal disease.

**Table 1:** Growth data from birth to time of presentation Weight-for-age percentiles were obtained from the Epic Electronic Health Record plot on the CDC’s standard growth chart for girls from birth to 36 months (see Figure 1). Weight gain (g) per day was calculated using the following formula: (current weight (g) – last weight (g))/days since last weight, and rounded to the nearest 10th CDC: Centers for Disease Control and Prevention

Age	Weight (kg)	Percentile for age (CDC, girls 0-36 months)	Weight gain (g) per day
Birth (at 37 weeks)	2.69	8.1%	
1 week	2.6	2.4%	-12
2 weeks	2.8	2.8%	28.5
1 month	3.4	7.1%	43
2 months	4.35	16.2%	34
2 months 2 weeks	4.6	13.6%	18
4 months	4.95	4.6%	8.5
4 months 2 weeks	5.65 (+0.695)	14.7%	50

Since her reported volume of intake was adequate, additional probing questions were asked about how her formula was prepared. It was discovered that the parents were using an automated formula machine to measure and distribute each of her bottles. Upon further history, the machine’s powder setting was set lower than recommended for her specific formula. This setting controls the grams of formula dispensed per ounce of water, thereby altering the concentration. Her parents noted that their formula machine was set at 2/3 instead of 5, which is recommended by the manufacturer for their specific commercial formula. This meant the prepared formula was less concentrated and provided significantly less than the recommended 20 kcal/oz, estimated to be 5 to 10 kcal less per oz. Even with the higher estimate of 15 kcal/oz, this would have resulted in approximately 160 kcal less per day at an intake of 32 oz/day.

Her parents were advised to manually fortify her formula to a higher concentration of 22 kcal/oz while continuing 5-oz feeds every four to six hours for a total of 32 oz/day. After two weeks, she reassuringly weighed 5.65 kg with an improved average weight gain of 50 g/day, returning her to the 14.72nd percentile. During this time, the family weighed the formula dispensed by their machine at the recommended setting of 5 and found it to be very close to the formula manufacturer’s recommended grams to provide 20 kcal/oz. The patient was ultimately able to resume her previous concentration of 20 kcal/oz and has continued to gain weight appropriately with no additional intervention, as shown in Figure 1.

## Discussion

While growth faltering has many causes, this report demonstrates a less common variation on the usual suspects, namely inadequate caloric intake secondary to incorrect formula mixing using consumer-grade, automated formula machines. Physicians and dietitians should counsel caregivers to carefully verify that machine settings are correct for the chosen formula. Previous studies, such as those by Rosenkranz et al., have reported that caregivers tend to overconcentrate formula. However, this case shows that formula mixing machines do not entirely eliminate the possibility of human error in the preparation of baby formula [5]. Using focus group feedback, as Gilmore et al. did, to improve mixing instruction labels may increase formula preparation accuracy and reduce user error [6].

While this case demonstrates the accidental preparation of a low-calorie and overly dilute formula, similar setup errors in automated formula machines may also lead to an overly concentrated formula. While this may not lead to weight loss, excessively high or incorrect concentrations of formula can still pose health risks in the form of electrolyte derangements. Multiple cases of hypernatremia from highly concentrated formula have been reported, with patients developing severe complications, including status epilepticus and cerebral hemorrhage [7,8]. While this is a rare occurrence, these cases further highlight the importance of counseling patients about proper formula concentration and machine settings.

Problems with automated formula machines have not been extensively described in the scientific literature, but news media outlets have highlighted consumer reports of inaccurate dispensing. There have also been complaints regarding formula machine use associated with other cases of growth faltering, and several product reviews reportedly mention inaccurate dispensing of formula [9]. Additionally, a study in the UK raised concerns that infant formula machines do not reach the proper temperature required to prevent bacterial contamination [10]. Further research on automated formula machines’ ability to accurately concentrate and safely prepare formula is needed to explore these issues. With these concerns in mind, providers must conduct a proper history and counsel families on the safe use of formula machines.

## Conclusions

This report demonstrates how crucial it is for physicians to (1) obtain a thorough pediatric nutrition history from families and (2) counsel caregivers on correct formula mixing methods to ensure adequate caloric intake, whether through manual or automated mixing methods. For families using automated machines, providers should consider confirming that machine settings are appropriate for the specific formula. Settings can be verified by weighing the formula powder dispensed by machines and comparing it to the formula manufacturer’s recommended scoop. When new patients present with suboptimal growth, inappropriate formula preparation, particularly with automated machines, should be considered a potential etiology and evaluated further. As these machines increase in popularity, providers must work closely with families to ensure proper nutrition and growth.
